# A mechanistic study on the repair of cadmium-induced male infertility using Yishen Tongluo formula based on network toxicology and experimental validation

**DOI:** 10.3389/fendo.2026.1837502

**Published:** 2026-06-04

**Authors:** Jing Hu, Yifei Wang, Sicheng Ma, Heng Liu, Yinuo Zhang, Wenlin Yu, Yizhe Gao, Jun Lu, Chenming Zhang

**Affiliations:** 1 Henan University of Chinese Medicine, Zhengzhou, China; 2 Auckland Bioengineering Institute, University of Auckland, Auckland, New Zealand

**Keywords:** cadmium-induced male infertility, hypothalamic-pituitary-gonadal axis, network toxicology, oxidative stress, sperm DNA fragmentation, testicular function, TP53, Yishen Tongluo formula

## Abstract

**Objective:**

This study employs network toxicology to screen for toxicological targets of cadmium chloride-induced male infertility, and validates the mechanism of Yishen Tongluo Formula in treating cadmium-induced male infertility through animal experiments.

**Method:**

Employing network toxicology to search for potential target molecules in the ChEMBL database, and STITCH databases using ‘cadmium chloride’ as the keyword to identify potential targets. Using “male infertility” as the keyword, we searched for male infertility-related targets in the OMIM, PharmGKB, and GeneCards databases. The primary components of the Yishen Tongluo Formula were then input into the SwissTargetPrediction and PharmMapper databases to identify potential targets. Cadmium chloride, Yishen Tongluo Formula component targets, and male infertility-related disease targets were imported into Venny 2.1.0 to construct a protein-protein interaction (PPI) network by identifying intersections. Cadmium chloride-induced male infertility-related targets were imported into Metascape for Gene Ontology (GO) and KEGG pathway enrichment analysis. Molecular docking validation was performed, and a ‘compound-target-pathway’ network was constructed for result visualisation. In animal experiments, 33 mice were randomly divided into three groups: a control group, a cadmium chloride-exposed group (Cd group), and a Yishen Tongluo Formula group, with 11 mice per group. Cadmium chloride was diluted in purified water to a working solution of 0.3 mg/ml. Except for the control group, the Cd group and Yishen Tongluo Formula group received cadmium chloride via gavage at 10 ml/kg for 70 days. After successful modeling, from day 31 onward, the cadmium-exposed group and the herbal intervention group received daily gavage of 10 ml/kg cadmium chloride working solution at 8:00 a.m. At 6:00 pm daily, the herbal intervention group received 10 ml/kg of the Yishen Tongluo Formula decoction solution via protective gastric lavage, The Yishen Tongluo Formula group received 1.2 g/ml of the decoction solution via gastric lavage, while the toxic exposure group received the same volume of deionised water. The control group received the same volume of deionised water at the same time points. All three groups underwent continuous gastric lavage for 40 days. Following organ collection, the following parameters were assessed: sperm DNA fragmentation index (DFI), testicular index, testicular superoxide dismutase (SOD), malondialdehyde (MDA), adenosine triphosphate (ATP), nitric oxide (NO), and serum follicle-stimulating hormone (FSH), luteinizing hormone (LH), and testosterone (T) were assessed.

**Conclusion:**

This study demonstrates that cadmium chloride induces male reproductive toxicity by targeting CFTR, SLC26A3, and SLC12A1 via the pancreatic secretion pathway, causing oxidative stress, endocrine disorders, sperm DNA damage, and testicular tissue injury. Yishen Tongluo Formula exerts therapeutic effects against cadmium-induced male infertility mainly by targeting TP53, which significantly improves testicular coefficient, reduces sperm DFI, enhances sperm quality, alleviates testicular oxidative stress, and restores the balance of reproductive hormones. These findings reveal the multi-target mechanism of Yishen Tongluo Formula in repairing cadmium-induced reproductive damage, provide a novel experimental basis for the treatment of heavy metal-related male infertility with traditional Chinese medicine, and suggest that further exploration of molecular mechanisms and clinical transformation will help develop safer and more effective intervention strategies for environmental toxin-induced reproductive dysfunction.

## 1 Introduction

Cadmium (Cd) is a toxic heavy metal present in various environments and substances in daily life, such as polluted air, water sources, soil, and certain foods. In recent years, with the intensification of environmental pollution, the harm caused by the heavy metal cadmium (Cd) to male reproductive health via dietary intake, smoking, and occupational exposure has become increasingly evident (1). Cadmium is a recognized carcinogen with proven mutagenic and genotoxic activity (2). Humans primarily encounter cadmium through the consumption of contaminated food and water, whilealso being exposed to a significant extent via inhalation of air pollution and smoking. Cadmium exhibits bioaccumulation in animals and plants, with a biological half-life of 25–30 years (3). Acute cadmium chloride exposure can cause significant reproductive system damage by exacerbating oxidative stress, inducing histopathological alterations (such as necrosis and oedema), triggering germ cell apoptosis, and causing sperm damage (reduced motility and concentration, increased abnormal sperm cells). Cadmium disrupts the hypothalamic−pituitary−gonadal (HPG) axis and interferes with endocrine signaling, leading to imbalanced secretion of luteinizing hormone (LH), follicle−stimulating hormone (FSH), and testosterone (T), which further impairs spermatogenesis and testicular function (1). Recent studies have demonstrated that cadmium induces severe genotoxicity and apoptosis in spermatogenic cells, which are key pathological events in male reproductive injury (4, 5). Research indicates that cadmium toxicity is closely associated with severe damage to multiple organs in both humans and animals, particularly the testes. In rat models, cadmium induces severe testicular atrophy, damage to the seminiferous tubules, and necrosis, thereby impairing reproductive capacity (6). The reproductive toxicity of cadmium in relation to male infertility was also verified in this study through network toxicology methods and animal experiments.

With the continuous advancement of the times, the incidence of male infertility has been increasing year by year. Research indicates that 30% to 50% of infertility cases are attributable to male factors (7). However, in terms of treatment, modern medical approaches to cadmium-induced reproductive damage—such as antioxidants and hormone replacement therapy—can alleviate certain symptoms. Nevertheless, these interventions are limited by their single-target nature, significant side effects, and inability to reverse long-term cumulative damage (8). Against this backdrop, traditional Chinese medicine has gradually emerged as a research focus within the field of reproductive medicine, owing to its unique advantages of holistic regulation and multi-targeted intervention (9). The current global reassessment of the value of natural medicines in chronic disease intervention, coupled with the standardized research on the material basis of classical formulas outlined in the Chinese Pharmacopoeia, has laid both theoretical and practical foundations for exploring the mechanisms and clinical application of the Yishen Tongluo Formula (10).

The Yishen Tongluo Formula is an empirical prescription with over two decades of clinical application. It possesses the efficacy of warming the kidneys and replenishing essence, as well as invigorating blood circulation and unblocking meridians. Research indicates that this formula demonstrates marked therapeutic efficacy in treating sperm DNA damage (11). As a quintessential example of integrating traditional medicine with modern toxicology, compared to modern medical treatments for male infertility caused by cadmium poisoning, the traditional Chinese medicine compound formula ‘Yishen Tongluo Formula’ employs the therapeutic principle of ‘nourishing the kidneys and replenishing essence, unblocking meridians and detoxifying’. It exhibits multi-targeted antioxidant effects, repairing oxidative damage, regulating reproductive hormone levels, restoring hypothalamic-pituitary-gonadal axis function, repairing DNA damage, maintaining genetic material stability, and offering comprehensive therapeutic regulation with safety advantages (12). To further investigate the mechanism by which the Yishen Tongluo Formula restores male reproductive capacity impaired by cadmium toxicity, this study employed network toxicology methods. By identifying common targets between cadmium chloride and male infertility, we validated the toxic effects of cadmium chloride on male fertility and its primary mechanisms of action. Concurrently, a cadmium chloride-induced mouse model of reproductive impairment was established. Intervention with the Yishen Tongluo Formula was administered, and changes in sperm DNA fragmentation index (DFI), testicular index, testicular superoxide dismutase (SOD), malondialdehyde (MDA), adenosine triphosphate (ATP), nitric oxide (NO), and serum follicle-stimulating hormone (FSH), luteinizing hormone (LH), and testosterone (T). The study aimed to investigate the mechanism by which the Yishen Tongluo Formula restores reproductive function in cadmium-exposed mice.

## 2 Materials and methods

### 2.1 Network toxicology methods

#### 2.1.1 Yishen Tongluo formula and cadmium chloride target screening

Potential targets for cadmium chloride were identified by searching the ChEMBL and STITCH databases using ‘cadmium chloride’ as the keyword. Subsequently, the obtained target data were merged and duplicates removed to establish the final target set for cadmium chloride. The primary constituents of the Yishen Tongluo Formula were imported into the SwissTargetPrediction database (https://swisstargetprediction.ch/) and the PharmMapper database (https://www.lilab-ecust.cn/pharmmapper/) to identify potential targets. Subsequently, the obtained target data were consolidated and duplicates removed to definitively establish the target set for the primary constituents of the Yishen Tongluo Formula.

#### 2.1.2 Screening of disease-related targets in male infertility

Using the keywords ‘male infertility’ and ‘renal injury’, searches were conducted across three disease target databases—OMIM, PharmGKB, and GeneCards—to identify targets associated with male infertility. Following consolidation and deduplication of the search results, these were used as functional targets relevant to male infertility.

#### 2.1.3 Protein-Protein Interaction (PPI) network analysis

Import the cadmium chloride component target and male infertility disease-related targets into Venny 2.1.0 (https://bioinfogp.cnb.csic.es/tools/venny/) to obtain the intersection. Submit the intersecting target nodes to the STRING database (https://string-db.org), selecting Homo sapiens as the species. Obtain the protein-protein interaction (PPI) network model. Import the PPI network results into Cytoscape 3.10.3 software and use the “Analyze Network” function in the toolbar to derive the degree, betweenness centrality, and closeness centrality. Subsequently, model styles were adjusted based on target node degree values to identify key targets implicated in cadmium chloride-induced male infertility.

#### 2.1.4 GO and KEGG enrichment analysis

Import the relevant targets associated with cadmium chloride-induced male infertility into Metascape (http://metascape.org/gp/index.html), set the species to ‘H. sapiens’, and perform Gene Ontology (GO) and Kyoto Encyclopedia of Genes and Genomes (KEGG) enrichment analyzes respectively. The results were visualized via the CNSknowall platform(https://www.cnsknowall.com/) to obtain biological information on intersecting targets, thereby analysing the potential mechanisms by which cadmium chloride induces male infertility.

#### 2.1.5 Construction of compound-target-pathway networks

Import the cadmium chloride, key target points, and enriched pathway relationships into Cytosccape 3.10.3 software to construct a visualized network diagram of the ‘compound-target-pathway’ relationship.

#### 2.1.6 Molecular docking validation

Search for proteins in the PDB database to obtain protein structures; Obtain 3D or 2D structures of chemical compounds from PubChem. Import the compound structures and protein structures into the CB-Dock2 platform (https://cadd.labshare.cn/cb-dock2/index.php) for molecular docking and visualization.

### 2.2 Materials and methods for animal experiments

#### 2.2.1 Laboratory animals and grouping

Thirty-three male BLBC/a grade mice, aged 6 weeks, were supplied by Beijing Huafukang Biotechnology Co., Ltd. (Licence No.: SCXK(Jing)2024-0003). Animals were housed within a barrier system under the following conditions: temperature 22.5 °C, humidity 58.6%, 24-hour illumination. Mice had free access to water and standard feed (Huan Yu Biological SPF-grade maintenance diet) and drinking water (purified water). Feed and bedding were replaced regularly. Animals underwent one week of acclimatization prior to experimentation, were weighed, numbered according to body weight, and grouped using a random number table. Animal experiments and procedures were conducted in accordance with the requirements of the Ethics Committee of Henan Provincial Hospital of Traditional Chinese Medicine. Animal ethics approval number: HZY2024007077. Following one week of acclimatization, 33 mice were randomly assigned to three groups: a control group, a cadmium chloride-exposed group (Cd group), and a Yishen Tongluo Formula group, with 11 mice per group.

#### 2.2.2 Medicinal products

Yishen Tongluo Formula (Composition: Cuscuta seed 20 g, Epimedium herb 20 g, Prepared rehmannia root 10 g, Astragalus root 20 g, Salvia root 30 g, Cyathula root 10 g, Leech, scalded 6 g) was supplied by the Sanjiu Ready-to-Take Granule Pharmacy of Henan Provincial Hospital of Traditional Chinese Medicine. Preparation standards adhered to the Henan Provincial Specificati Yishen Tongluo Formulaons for Processing Chinese Herbal Medicinal Materials. The gastric lavage dosage of the Yishen Tongluo Formula was calculated according to relevant proportions (13), then decocted by the Chinese Medicine Preparation Room of Henan Provincial Hospital of Traditional Chinese Medicine into a concentrated compound solution at 1.2 g/ml. This was collected in sterile glass bottles and stored at 4 °C for future use.

#### 2.2.3 Reagent

Cadmium chloride was obtained from Yuanye Biotechnology Co., Ltd., Shanghai, China (Batch No.: 10108-64-2). Mouse ATP ELISA kit, mouse adenosine triphosphate synthase, luteinizing hormone, follicle-stimulating hormone, testosterone, NO content assay kit, SOD assay kit and MDA assay kit were purchased from Jiangsu Meimian Industrial Co., Ltd., Yancheng, China (Batch No.: ml824959, ml001984L, ml001910L, ml001948L, mlsh0411, mlsh0697, mlsh0387). Rabbit Anti-CLDN11 antibody, Rabbit Anti-Occludin antibody and Rabbit Anti-Connexin 43 antibody were purchased from Beijing Biosynthesis Biotechnology Co., Ltd., Beijing, China (Batch No.: BS-21509R, BS-10011R, BS-8987R).

#### 2.2.4 Instrument

100-mesh BS-100-XBS cell filter (Biosharp, Beijing, China); XPR226DR/AC electronic analytical balance (Mettler Toledo, Zurich, Switzerland); Fresco21 high-speed low-temperature centrifuge (Thermo Fisher Scientific, Waltham, MA, USA); Multifunctional microplate reader (Thermo Fisher Scientific, Waltham, MA, USA), Accari C6 flow cytometer (BD Biosciences, San Jose, CA, USA); WCJY-9000 fully automated semen analyzer (Beijing Weili Technology Co., Ltd., Beijing, China); JX-2016 homogeniser (Shanghai Jingxin Industrial Development Co., Ltd., Shanghai, China).

#### 2.2.5 Model preparation and drug administration

Dilute cadmium chloride in pure water to a working solution of 0.3 mg/ml. The dose of cadmium chloride (3 mg/(kg·d)) was selected based on previous studies showing it induces stable reproductive injury in mice without severe systemic toxicit (1, 14). Except for the blank group, both the Cd group and the Yishen Tongluo Formula group received oral gavage at 10 ml/kg for 70 days (15). At 30 days post-exposure, three mice were randomly selected from each group. Sperm from one epididymis was collected from each mouse to assess sperm DFI, thereby determining the success of modeling.

Following successful modeling, from day 31 onward, mice in both the cadmium-exposed group and the traditional Chinese medicine intervention group received 10 ml/kg of cadmium chloride working solution via gastric lavage daily at 8:00 am. At 18:00 daily, the Chinese herbal intervention group received 10 ml/kg of Yishen Tongluo Formula decoction via protective gavage. The Yishen Tongluo Formula group received 1.2 g/ml of the decoction. This dose was converted from the clinical adult dose according to the body surface area ratio between mice and human and had been verified effective in our previous studies (13). The poisoned group received the same dose of deionized water via gastric lavage, while the blank group received the same dose of deionized water at the same time. All three groups underwent continuous gastric lavage for 40 days.

#### 2.2.6 Testing parameters

##### 2.2.6.1 Serum hormone assay

Following completion of experiments, mice in each group were fasted for 12 hours. Anesthesia was induced via intraperitoneal injection of 4% diethyl ether (0.8 ml/100 g body weight). Blood was collected from the eyeball after anesthesia. Centrifugation was performed at 3,000 rpm for 10 minutes. Serum was collected and stored at -20 °C for subsequent measurement of reproductive hormones. Both testes and epididymides were excised and weighed. Testosterone (T), FSH, and LH were measured by ELISA following the manufacturer’s instructions and standard protocols (14, 15).

##### 2.2.6.2 Determination of testicular coefficient in mice

Promptly remove both testes, weigh them, and calculate the testicular coefficient. Testicular coefficient (%) = Testicular weight/Final body weight of mouse × 100%.

##### 2.2.6.3 Sperm DFI measurement

After washing the left epididymis in physiological saline, promptly place it in 1 ml of physiological saline. Use scissors to finely chop the tail of the epididymis, mix thoroughly, and incubate in a 37 °C water bath for 30 minutes to prepare a sperm suspension for DFI analysis. Sperm DNA fragmentation index (DFI) was measured using the sperm chromatin dispersion (SCD) assay following the manufacturer’s instructions and previously reported methods (1, 16).

##### 2.2.6.4 Test for indicators of testicular oxidative stress

Take mouse testes, accurately weigh the testicular mass, add 0.9% sodium chloride solution at a ratio of body weight (g): volume (mL) = 1:9, homogenize in an ice-water bath to prepare a 10% tissue homogenate, centrifuge at 3,500 r/min (centrifugal radius = 7.5 cm) for 10 minutes, and collect the supernatant for analysis. SOD, NO, MDA, glutathione (GSH), and total oxidative stress (TOS) levels in mouse testicular homogenate were measured following the kit instructions and standardized oxidative stress detection methods (4, 5).

##### 2.2.6.5 HE staining was employed to examine the histological morphology of testicular tissue in each group of mice

The fixed spleen and kidney tissues were embedded in paraffin, sectioned to prepare paraffin sections, and then deparaffinized and rinsed. HE staining was performed using a commercial HE staining kit following standard histological protocols (3, 14). Subsequent steps included dehydration, clearing, and mounting. Tissue morphology was observed under a light microscope (×400 magnification), and images were captured.

##### 2.2.6.6 Statistical methods

Data analysis was conducted using SPSS 25.0 statistical software (IBM Corp., Armonk, NY, USA). Count data were analyzed using the chi-square test. For continuous data meeting the assumption of normal distribution, tests for homogeneity of variance were performed. Where variances were homogeneous, one-way analysis of variance was conducted. Non-parametric tests were employed for data failing normality or homogeneity of variance. Ordinal data were analyzed using the Mann-Whitney U test. A p-value < 0.05 was considered statistically significant.

## 3 Results

### 3.1 Results of network toxicology

#### 3.1.1 Predicting targets for the treatment of cadmium chloride-induced male infertility using the Yishen Tongluo formula based on network toxicology techniques

**Figure 1:**
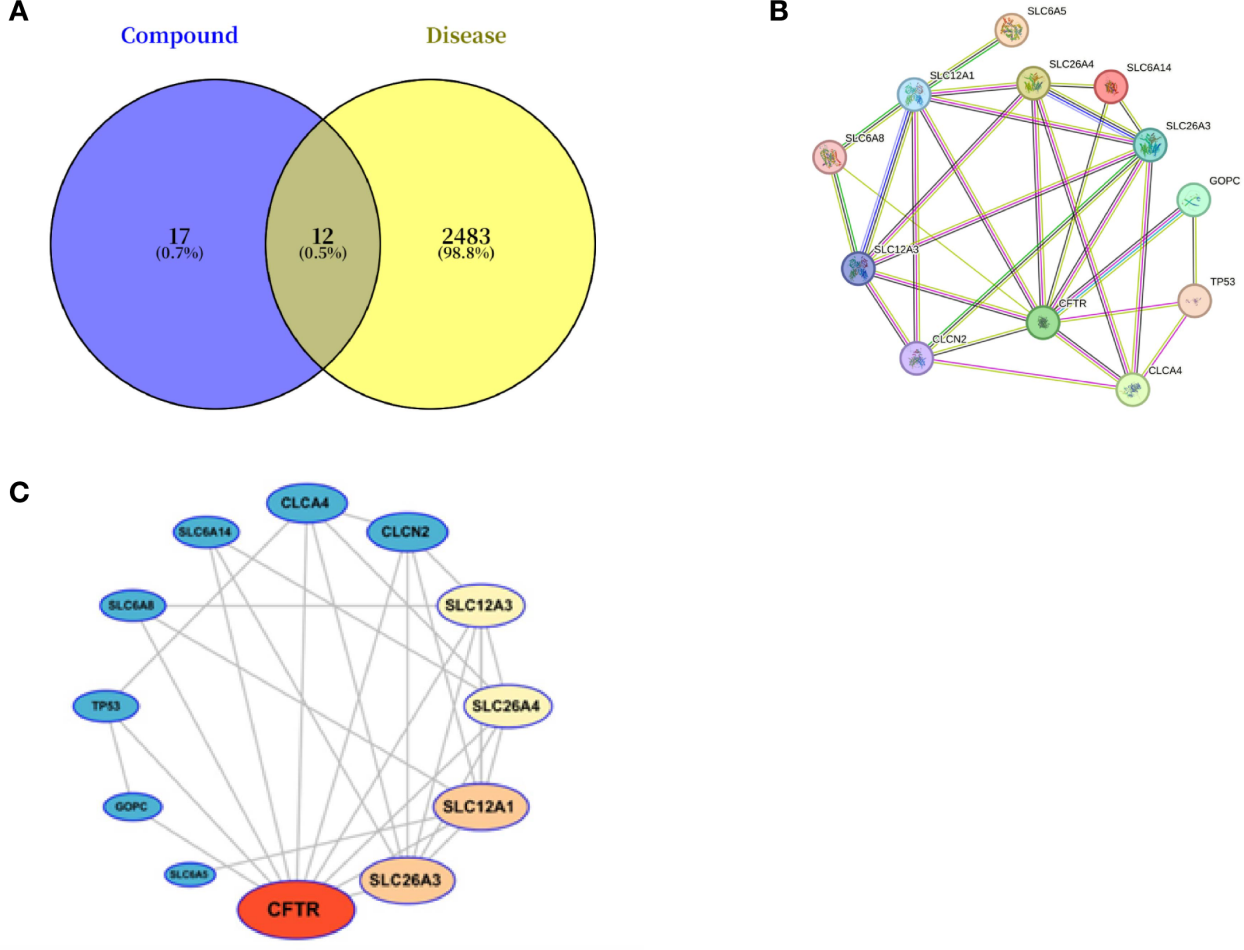
Identification of common targets and network analysis of cadmium chloride and male infertility. **(A)** Common targets of cadmium chloride and male infertility. **(B)** Protein-protein interaction (PPI) network of cadmium chloride and male infertility. **(C)** Target interaction network.

Target identification for cadmium chloride was conducted via the ChEMBL and STITCH databases, yielding a combined total of 29 action targets. Concurrently, male infertility-related targets were retrieved from the OMIM, PharmGKB, and GeneCards databases. The GeneCards database was filtered based on a Relevance score ≥10, resulting in 2,495 relevant targets. The male infertility-related targets and cadmium chloride toxicity targets were imported into Venny 2.1.0 software for intersection analysis, yielding 12 cadmium chloride-induced male infertility targets (**Figure 1A**). Target identification for the seven primary constituents of the Yishen Tongluo Formula was conducted using PharmMapper and SwissTargetPrediction. The combined analysis yielded 256 action targets for the Yishen Tongluo Formula.

#### 3.1.2 PPI network construction and analysis

The 12 intersecting target genes identified through screening were imported into the STRING database to generate a protein interaction network diagram linking cadmium chloride with male infertility, as shown in **Figure 1B**. The resulting data were exported to Cytoscape 3.10.3 software for protein-protein interaction (PPI) network analysis. The degree values of the targets were obtained using the “Analyze Network” function in the toolbar. Subsequently, the model style was adjusted based on these degree values to identify key targets implicated in cadmium chloride-induced male infertility. By setting thresholds of Degree ≥ 7, Betweenness Centrality (BC) ≥ 0.05, and Closeness Centrality (CC) ≥ 0.73, three critical targets were identified: CFTR, SLC26A3, and SLC12A1. Node size and color intensity were adjusted according to Degree values to construct the model. This yielded three key targets: CFTR, SLC26A3, and SLC12A1. Node size and color intensity were scaled according to degree values to construct the PPI network diagram (**Figure 1C**). These core targets represent potential key sites in cadmium-induced male infertility, playing a primary role in the pathological process.

#### 3.1.3 GO enrichment analysis and KEGG pathway analysis of cadmium chloride-induced male infertility targets

**Figure 2:**
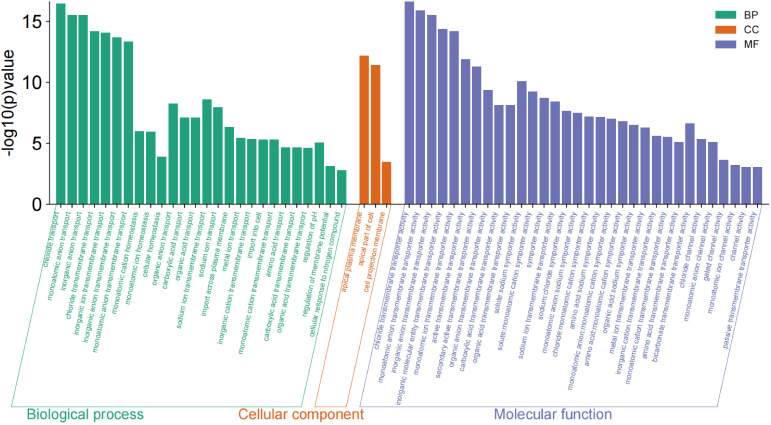
GO enrichment analysis bar chart.

The 12 intersecting targets implicated in cadmium chloride-induced male infertility were imported into Metascape. Following submission, species selection was set to ‘H. sapiens’ with P < 0.01. GO functional enrichment analysis was conducted, with results visualized via the CNSknowall platform to generate **Figure 2**. The GO analysis encompassed three domains: Biological Process, Cellular Component, and Molecular Function. Results indicated that the intersecting genes were enriched across 26 biological processes, primarily involving transmembrane transport, regulation of membrane potential, and cellular response to nitrogen compounds; Enrichment occurred in three cellular components, chiefly apical plasma membrane, cell apex, and cell process membrane; enrichment was observed in 32 molecular function-related processes, primarily involving ion transport across membranes, organic molecule transport across membranes, active transport mechanisms, channel activity, and transporter function. The 12 intersecting targets were imported into Metascape. Following submission, species selection was set to ‘H. sapiens’ with P < 0.01. KEGG pathway analysis was conducted, identifying one KEGG pathway. The analysis results indicated the pancreatic secretion pathway.

#### 3.1.4 Construction of the compound-target-pathway network

**Figure 3:**
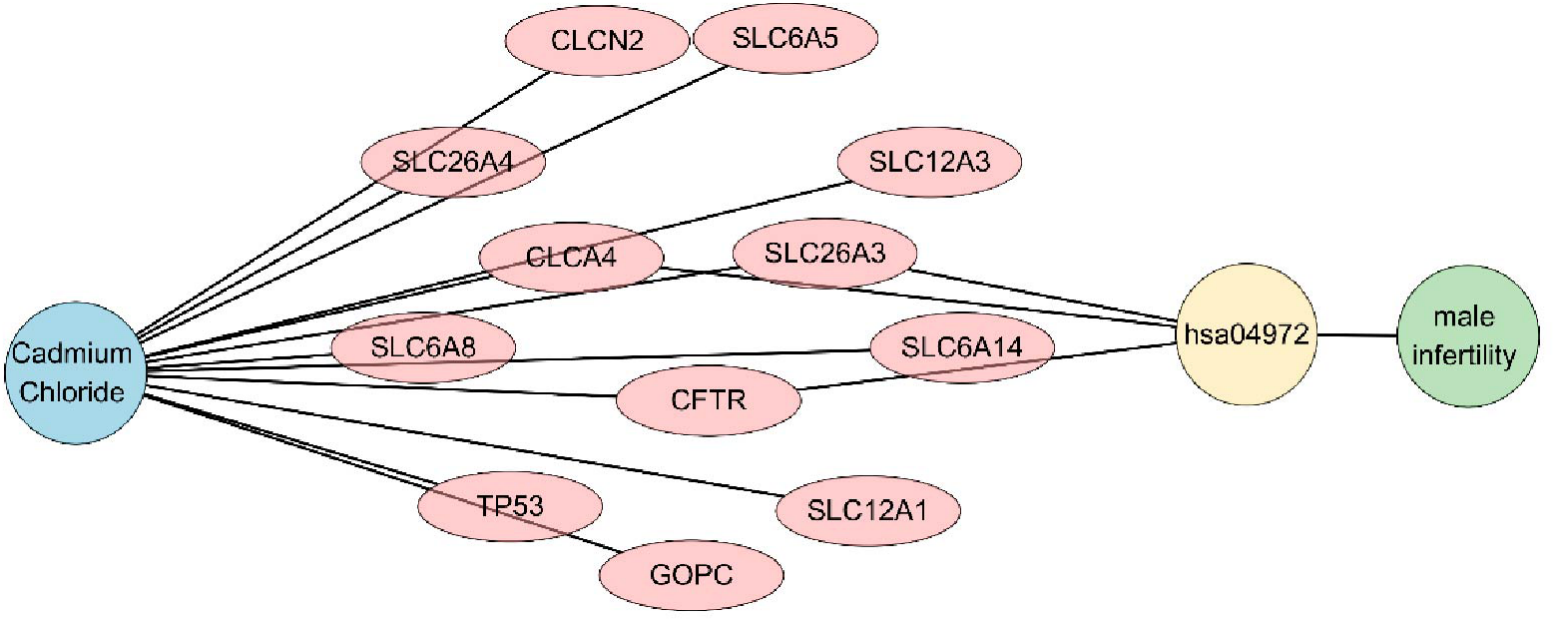
Compound-target-pathway network diagram.

Twelve targets implicated in cadmium chloride-induced male infertility were identified. The network and type files were organized and imported into Cytosccope software to generate a ‘compound-target-pathway’ network diagram, as shown in **Figure 3**.

#### 3.1.5 Docking validation of the active ingredient causing male infertility due to cadmium chloride with target molecules

**Figure 4:**
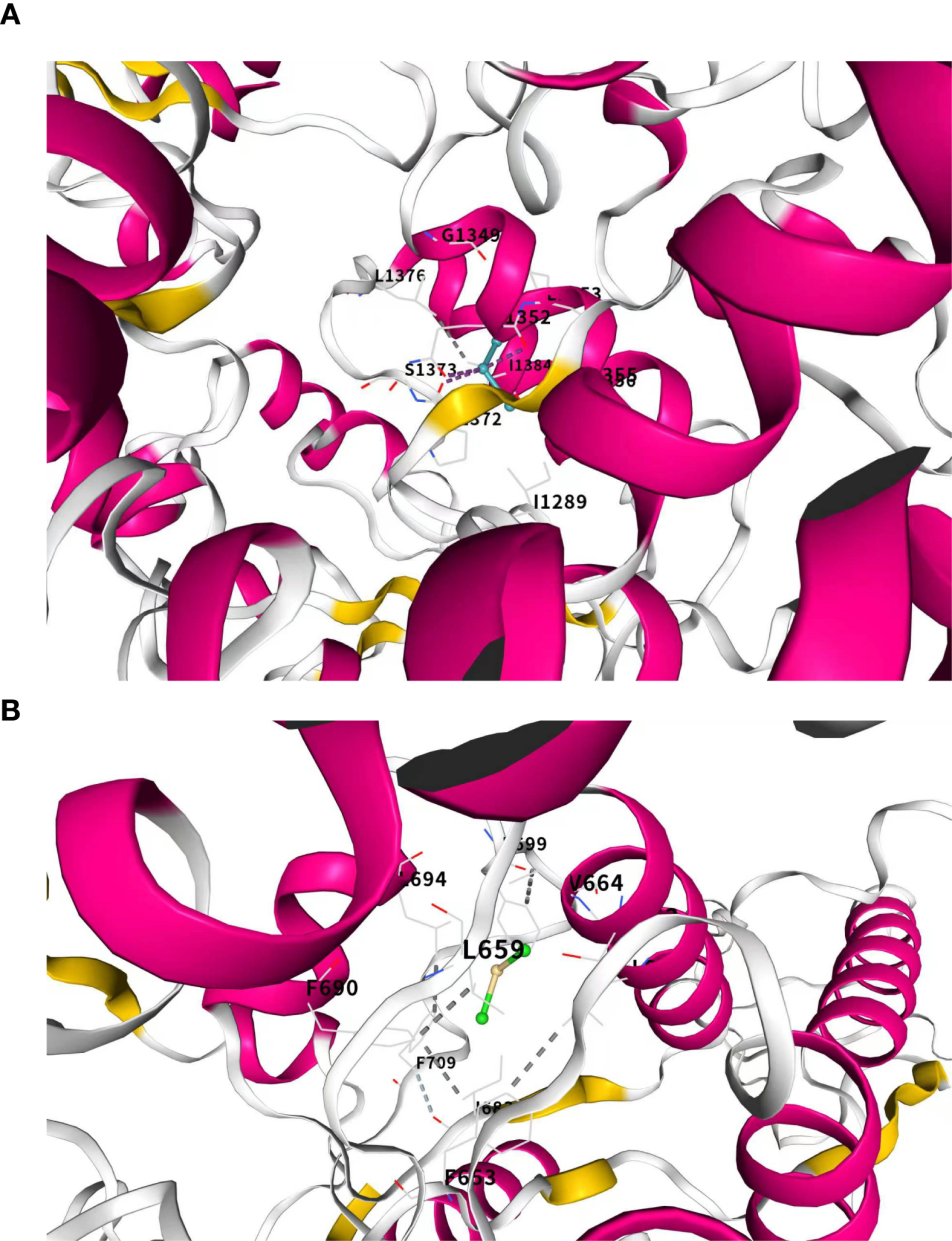
Molecular docking of the core target with cadmium chloride **(A)** CFTR and cadmium chloride **(B)** SLC26A3 and cadmium chloride.

Molecular docking was performed between the two core proteins identified via PDB database searches and cadmium chloride. Compound structures and protein structures were imported into CB-Dock2 to obtain docking results. The more stable the conformation of the ligand-receptor complex, the lower the binding energy required. The docking results indicated that the minimum binding energies between molecules and target proteins were all <0, demonstrating that both ligands and receptors could bind spontaneously. For CFTR molecular docking with cadmium chloride, the most stable docking structure C1 was visualized based on binding energy. Similarly, for SLC26A3 molecular docking with cadmium chloride, the most stable docking structure C1 was visualized according to binding energy. Visualization results for both are presented in **Figure 4**.

#### 3.1.6 Predicting targets for Yishen Tongluo formula in treating cadmium chloride-induced male infertility using network pharmacology techniques

**Figure 5:**
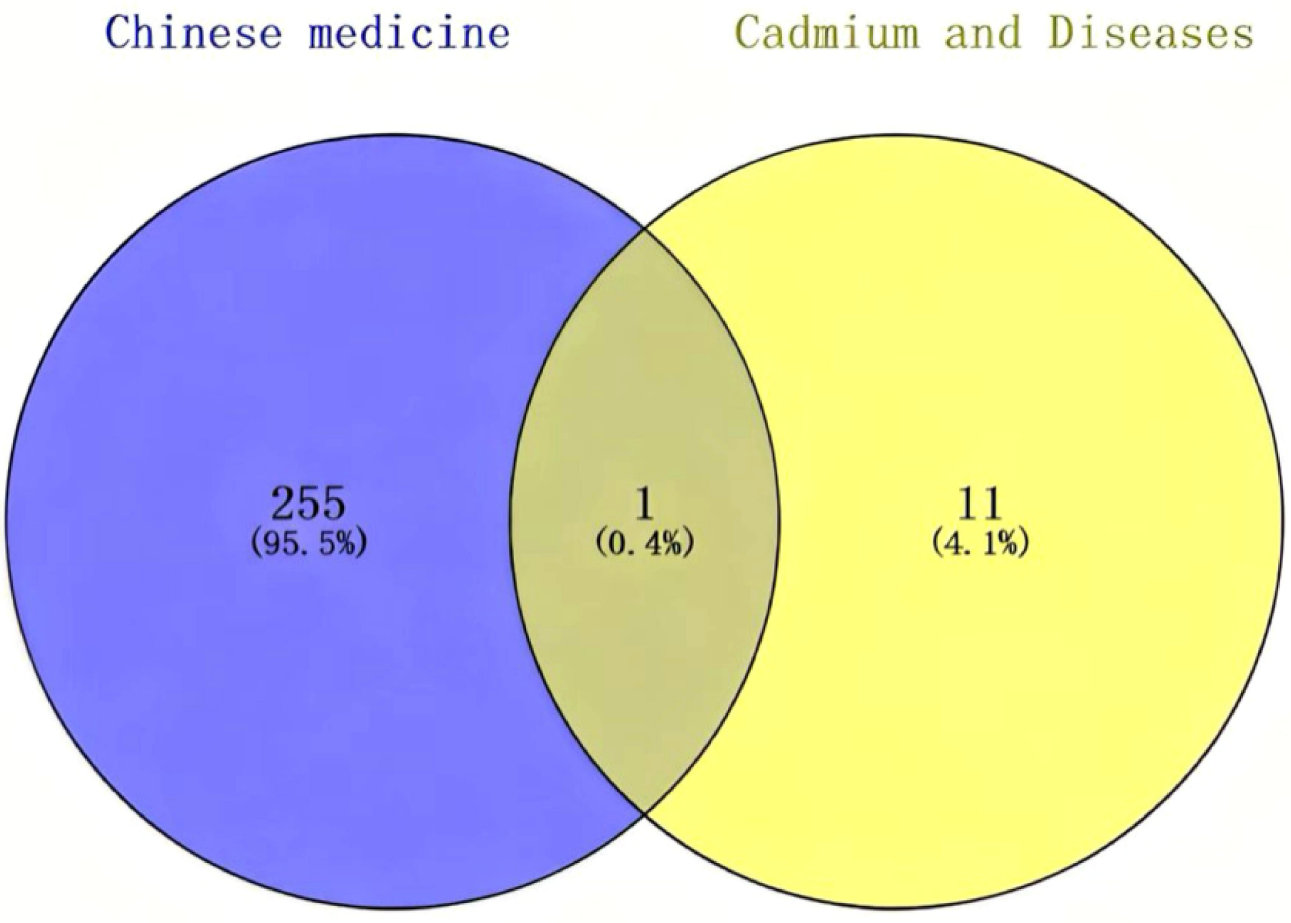
Common targets of the Yishen Tongluo formula and cadmium chloride-induced male infertility.

The primary components of the Kidney-Benefiting and Meridian-Unblocking Formula were input into Venny 2.1.0 software alongside 12 common targets associated with cadmium chloride-induced male infertility. This yielded one shared target, TP53, as shown in **Figure 5**.

### 3.2 Results of animal experiments

#### 3.2.1 Comparison of testicular coefficients among groups of mice

See **Table 1**. Compared with the blank group, the testicular coefficient was significantly reduced in the cadmium chloride-exposed group (P < 0.05). Compared with the model group, the testicular coefficient increased in the Yishen Tongluo Formula group, with statistically significant differences observed in all comparisons (P < 0.05).

**Table 1:** Comparison of testis coefficients among groups (x̄ ± s, n = 8, %).

Group	Testicular coefficient
Blank group	0.81 ± 0.03
Model Group	0.51 ± 0.09^*^
Yishen Tongluo Formula	0.76 ± 0.10^△^

*P<0.05 vs. blank group; ^△^P <0.05 vs. model group.

#### 3.2.2 Comparison of sperm DFI among groups of mice

See **Table 2**. Compared with the blank group, the DFI levels in the toxin-exposed group were significantly elevated (P < 0.05). Compared with the model group, the DFI levels in the Yishen Tongluo Formula group were significantly reduced (P < 0.05).

**Table 2:** Comparison of sperm DFI among groups (x̄ ± s, n = 8, %).

Group	DFI
Blank group	5.04 ± 1.34
Model Group	9.80 ± 11.91^*^
Yishen Tongluo Formula	5.89 ± 0.89^△^

*P<0.05 vs. blank group; ^△^P <0.05 vs. model group.

#### 3.2.3 Comparison of serum hormones LH, FSH, and T among groups of mice

See **Table 3**. Compared with the blank group, serum LH, FSH, and T levels were significantly reduced in the model group (P < 0.05). Compared with the model group, serum LH, FSH, and T levels were significantly elevated in the Yishen Tongluo Formula group (P < 0.05).

**Table 3:** Comparison of serum hormones LH, FSH, and T among groups of mice (x̄ ± s, n = 8, %).

Group	LH (pg/ml)	FSH (ng/ml)	T (pg/ml)
Blank group	560.76	12.84	427.428
Model Group	409.508^*^	8.65^*^	177.429^*^
Yishen Tongluo Formula	468.43	11.23^△^	255.953^△^

*P<0.05 vs. blank group; ^△^P <0.05 vs. model group.

#### 3.2.4 Comparison of testicular oxidative markers MDA, SOD, and NO among mouse groups

See **Table 4**. Compared with the blank group, the model group exhibited significantly elevated MDA and NO levels (P < 0.05) and markedly reduced SOD activity (P < 0.05). Compared with the model group, the Yishen Tongluo Formula group demonstrated significantly decreased MDA and NO levels (P < 0.05) and markedly increased SOD activity (P < 0.05).

**Table 4:** Comparison of testicular oxidative factors MDA, SOD, and NO among mouse groups (x̄ ± s, n = 8, %).

Group	MDA (nmol/mL)	SOD (U/mg)	NO (μmol/L)
Blank group	11.87 ± 0.18	616.42 ± 50.84	0.87 ± 0.03
Model Group	15.29 ± 0.11^*^	456.29 ± 44.39^*^	1.15 ± 0.03^*^
Yishen Tongluo Formula	14.38 ± 0.11	565.87 ± 31.93^△^	0.88 ± 0.04^△^

*P<0.05 vs. blank group; ^△^P <0.05 vs. model group.

#### 3.2.5 Analysis of H&E-stained sections

**Figure 6:**
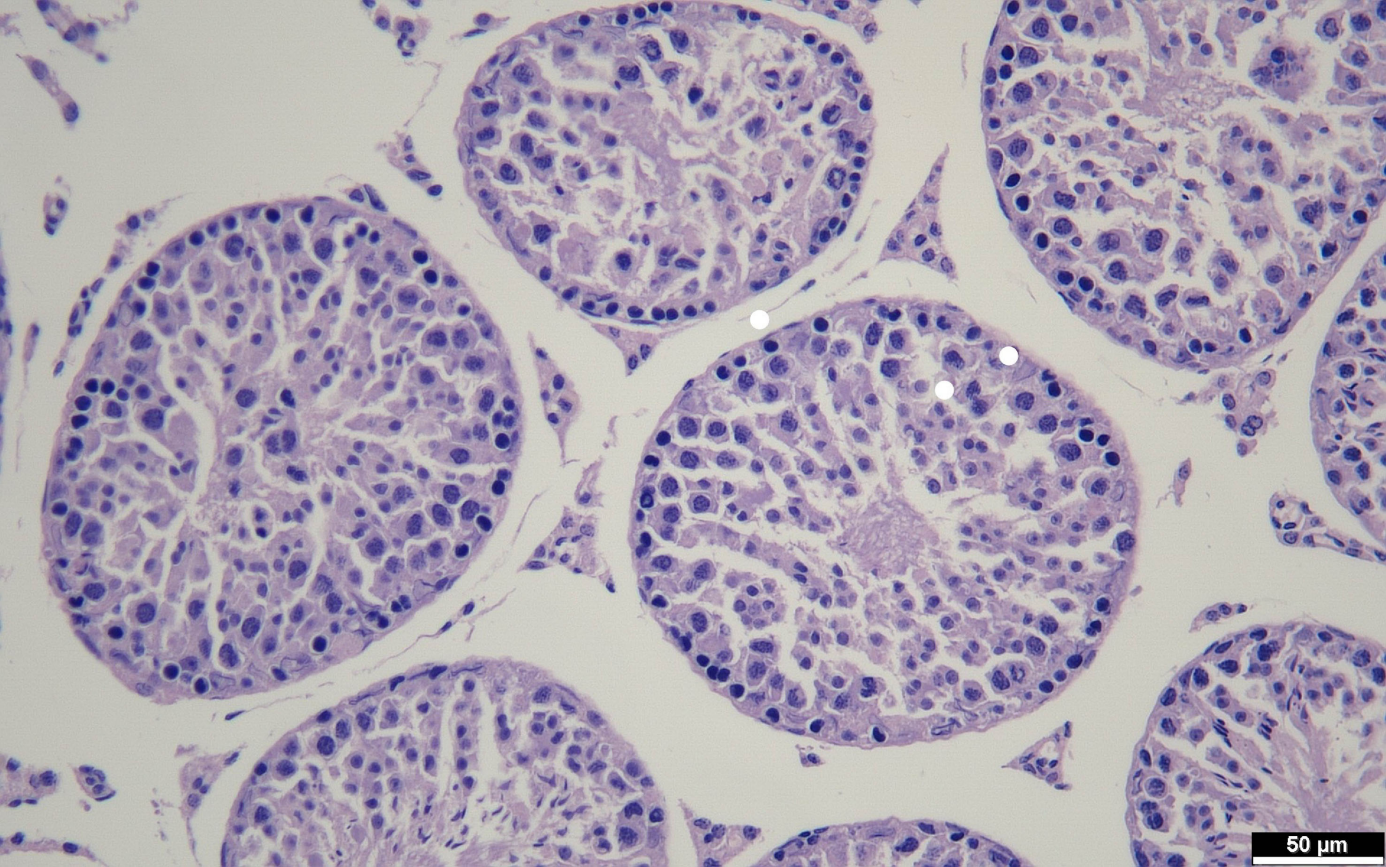
Hematoxylin and eosin staining of testicular tissue in the cadmium chloride-exposed group (×400 magnification, 50 μm). The reduction or absence of spermatogenic epithelium, disordered seminiferous tubule structure, increased testicular interstitial volume, enlarged seminiferous tubule spaces, loss of seminiferous epithelium with intraepithelial vacuolation, and significantly reduced number of germ cell layers were observed.

**Figure 7:**
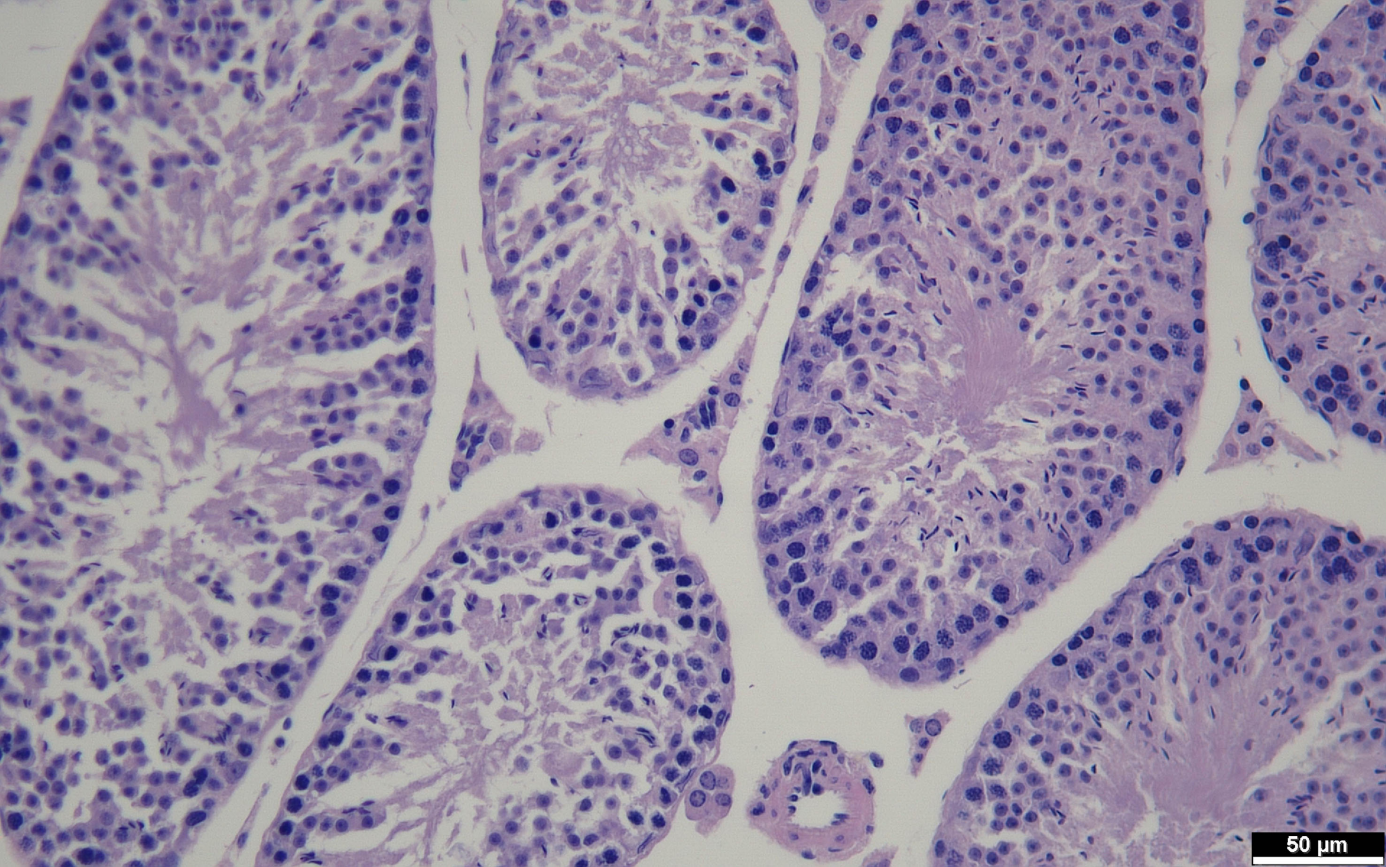
Hematoxylin and eosin staining of testicular tissue in the Yishen Tongluo formula-treated group (×400 magnification, 50 μm). The histopathological damage of testicular tissue was significantly improved, with reduced seminiferous tubule space, restored seminiferous epithelium, and increased number of spermatogenic cells compared with the cadmium chloride-exposed group.

**Figure 8:**
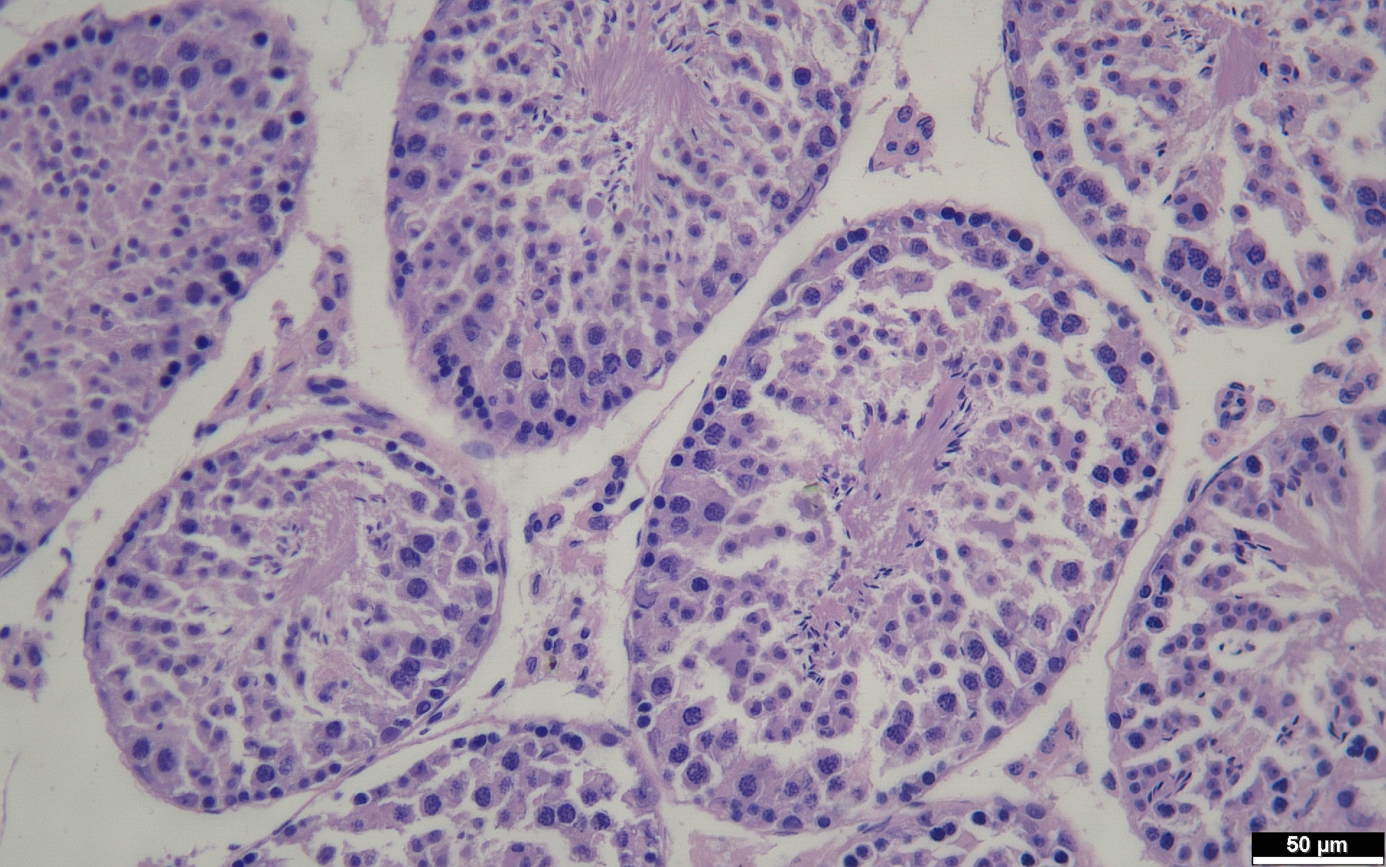
Hematoxylin and eosin staining of testicular tissue in the blank control group (×400 magnification, 50 μm). The testicular tissue showed a normal histological structure with neatly arranged seminiferous epithelium and spermatogenic cells at all stages, uniform nuclear membrane granule staining, and consistent cytoplasmic staining.

HE staining revealed that the control group exhibited normal histological structure of testicular tissue, The diameter of seminiferous tubules and Johnsen score were quantitatively analyzed to evaluate testicular injury and repair effect (17, 18) Nuclear membrane granules displayed uniform staining intensity, and cytoplasmic staining was consistent throughout. In the cadmium chloride-exposed group, increased testicular interstitial volume led to enlarged seminiferous tubule spaces, with tubular abnormalities observed. Detached cells were found within the seminiferous tubules, accompanied by loss of the seminiferous epithelium and intraepithelial vacuolation. The number of cell layers and germ cells within the seminiferous tubule lumens decreased significantly. Compared to the normal control group, the cadmium chloride-exposed group exhibited marked differences in testicular histology, with the seminiferous epithelium showing severe cellular loss, vacuolation, disorganized germ cell arrangement, and necrotic seminogenesis impairment (**Figure 6**). These pathological changes are closely related to excessive oxidative stress, germ cell apoptosis and inflammatory injury induced by cadmium (1, 14). Immunohistochemical analysis was used to detect apoptosis cascade (Caspase-3, Bax/Bcl-2) and NF−κB signaling pathway (14). Compared with the cadmium chloride-exposed group, histopathological damage in the testicular tissue of mice treated with the Yishen Tongluo Formula was significantly alleviated, indicating that Yishen Tongluo Formula effectively protects testicular tissue structure from cadmium-induced injury (**Figure 7**). Normal testicular tissue histology of the blank control group was exhibited as the reference (**Figure 8**).

## 4 Discussion

In the current treatment of male infertility, modern medical approaches targeting cadmium-induced reproductive damage—such as antioxidants and hormone replacement therapy—can alleviate certain symptoms. However, these interventions are limited by their single-targeted nature, significant side effects, and inability to reverse long-term cumulative damage (1). To further investigate the mechanism by which the Yishen Tongluo Formula restores male reproductive capacity impaired by cadmium toxicity, this study employed network toxicology and pharmacology methods combined with animal experiments. By identifying common targets between cadmium chloride and male infertility, we validated the toxic effects of cadmium chloride on male infertility and its primary mechanisms of action. Concurrently, a cadmium chloride-induced mouse model of reproductive impairment was established. Intervention with the Yishen Tongluo Formula was administered to observe changes in sperm DNA fragmentation index (DFI), testicular index, testicular superoxide dismutase (SOD), malondialdehyde (MDA), adenosine triphosphate (ATP), nitric oxide (NO), and serum follicle-stimulating hormone (FSH), luteinizing hormone (LH), and testosterone (T). This study innovatively combined network toxicology and animal experiments to reveal the multi-target mechanism of Yishen Tongluo Formula against cadmium-induced reproductive damage, which provides a novel natural medicine strategy for heavy metal-related male infertility (17, 18). This study employed network toxicology to conduct target searches for cadmium chloride via databases, yielding a consolidated list of 29 action targets. Concurrently, a database search for male infertility-related targets yielded 2,495 relevant targets after screening. These male infertility-related targets and cadmium chloride toxicity targets were imported into Venny 2.1.0 software for intersection analysis, yielding 12 cadmium chloride-induced male infertility targets. These 12 intersecting targets were then imported into the STRING database to generate a protein interaction network diagram linking cadmium chloride and male infertility. Post-screening, three key targets emerged: CFTR, SLC26A3, and SLC12A1. The 12 cadmium chloride-induced male infertility target intersections were then analyzed in Metascape for Gene Ontology (GO) enrichment and KEGG pathway studies. This revealed cadmium chloride primarily exerts functional effects through transmembrane transport, apical plasma membrane, and ion transport activities, predominantly impacting male reproductive function via the pancreatic secretion pathway. Subsequently, results were visualized through constructing a ‘compound-target-pathway’ network and molecular docking validation. Network toxicology research offers potential for elucidating the scientific mechanisms underlying cadmium chloride-induced male infertility, providing scientific reference for the clinical application of Yishen Tongluo Formula in treating cadmium-induced decline in male reproductive capacity.

Male fertility is closely linked to the integrity of sperm DNA. Should sperm DNA become damaged due to various factors, it diminishes sperm quality and weakens a man’s reproductive potential (16). Additionally, cadmium may elevate sperm deformity rates through epigenetic alterations (such as abnormal DNA methylation) and direct damage to sperm DNA (14). The Yishen Tongluo Formula reduces DNA fragmentation by regulating long non-coding RNAs (lncRNAs) and DNA repair-associated proteins such as XRCC1 and OGG1 (19). Experiments demonstrated that following intervention with the Yishen Tongluo Formula, rats exhibited a significant reduction in DFI and marked restoration of abnormal methylation in genes encoding both mRNA and lncRNA (20). Compared to traditional medications (such as antioxidants or hormone replacement therapy) with their single-target approach and associated risks of side effects, the Yishen Tongluo Formula employs the therapeutic principle of ‘nourishing the kidneys and replenishing essence, unblocking meridians and detoxifying’. Different from single-target antioxidants, Yishen Tongluo Formula exerts multi-dimensional protective effects through anti-oxidation, DNA repair, and improving spermatogenic cell ultrastructure, which is the unique advantage of this prescription (21). Its compound constituents (such as Salvia miltiorrhiza and Hirudo nipponica) may improve testicular microcirculation. Concurrently, acute toxicity studies indicate that the maximum tolerated dose of the Yishen Tongluo Formula is 300 g/kg (equivalent to 181 times the clinical dose), with no evidence of hepatic or renal impairment observed (22). Clinical studies indicate that this formula achieves an overall efficacy rate of 92.5% in treating idiopathic asthenozoospermia, with sperm DNA integrity improving from 63.11% to 80.26% (23). This experiment established a mouse model of sperm DNA damage through cadmium exposure. Data revealed that compared with the blank control group, cadmium exposure significantly elevated the sperm DNA fragmentation index (DFI) levels in mice, while the testicular index exhibited a downward trend. Following intervention with traditional Chinese medicine, the sperm DNA fragmentation index decreased, and the testicular index showed an upward trend.

In addition to pathological damage, oxidative stress and ultrastructural damage to spermatogenic cells are core mechanisms of cadmium-induced reproductive toxicity. Cadmium destroys mitochondrial structure and induces oxidative stress, further leading to reproductive dysfunction (18, 24). Cadmium exposure induces excessive reactive oxygen species (ROS) accumulation in testicular tissue, which further destroys sperm DNA integrity and mitochondrial function (25). The Yishen Tongluo Formula significantly alleviates oxidative stress damage by enhancing superoxide dismutase (SOD) and glutathione peroxidase (GSH-Px) activity while reducing malondialdehyde (MDA) and nitric oxide (NO) levels (26). Oxidative stress is one of the key factors causing sperm DNA damage. Under normal circumstances, seminal plasma contains antioxidant substances that protect sperm DNA. However, when the antioxidant capacity of seminal plasma diminishes, or when reactive oxygen species (ROS) are produced in excessive quantities beyond the antioxidant capacity of seminal plasma, sperm DNA becomes damaged. This subsequently leads to a decline in male sperm quality and reduced reproductive potential (27). Research indicates that Wang Zulong et al. (2017) noted the antioxidant effects of the Yishen Tongluo Formula are primarily attributable to the free radical scavenging capacity of constituents such as Salvia miltiorrhiza and Astragalus membranaceus (19). This experiment established a mouse model of sperm DNA damage through cadmium exposure. Data revealed that in the cadmium-exposed group, the activity of the antioxidant enzyme superoxide dismutase (SOD) in testicular tissue was significantly reduced, while levels of the peroxidation metabolite malondialdehyde (MDA) and nitric oxide (NO) were markedly elevated. This suggests that cadmium exposure induces the production of substantial reactive oxygen species (ROS) within the body. This leads to diminished SOD activity, resulting in oxidative damage and increased levels of the peroxidation metabolite MDA. Concurrently, elevated NO levels cause damage to the lipid membrane, further exacerbating oxidative stress injury (28).

Besides oxidative stress and DNA damage, endocrine disorder is also an important pathway for cadmium to impair male reproduction. Luteinizing hormone (LH) and follicle-stimulating hormone (FSH) are key hormones secreted by the pituitary gland (29, 30) Their secretion is regulated by the hypothalamus, which in turn is subject to negative feedback regulation by hormones secreted by the testes (31). The decline in endogenous testosterone (T) production is one cause of diminished male sexual function. Indicators such as FSH, LH, and testosterone (T) levels are influenced by factors including body weight, age, and lifestyle (32). The gonadotropins FSH and LH secreted by the pituitary gland, along with testosterone secreted by the testicular interstitial cells, exert their effects on spermatogenesis and maturation through distinct pathways. Concurrently, these hormonal indicators exhibit reciprocal relationships of either mutual promotion or inhibition (25, 33). The process of spermatogenesis in the testes is precisely regulated by the hypothalamic-pituitary-testicular endocrine axis. Any disruption in the balance of this axis may adversely affect spermatogenesis to varying degrees (34). Cadmium inhibits key enzymes in testosterone synthesis, reducing serum testosterone levels and disrupting spermatogenesis. The Yishen Tongluo Formula restores the physiological balance of gonadotropins (FSH, LH) and testosterone (T) by regulating the hypothalamic-pituitary-testicular axis (30). Research indicates that serum testosterone levels in cadmium-exposed rats recovered from 4.42 nmol/L to 4.96 nmol/L, while FSH and LH levels were significantly reduced (35). Sun Zixue et al. (2019) indicated that kidney-tonifying Chinese medicinal herbs such as dodder seed and epimedium possess hormone-like effects, capable of promoting testosterone synthesis (20). The experimental results indicate (36), following traditional Chinese medicine intervention, serum levels of LH, FSH, and T were elevated compared to the model group. The mechanism may involve promoting spermatogenesis by stimulating pituitary secretion of FSH and LH and enhancing testicular secretion of T. Additionally, it may stabilize endogenous sex hormone levels by regulating the hypothalamic-pituitary-testicular axis, reducing cadmium’s inhibitory effect on the hypothalamic-pituitary axis, elevating LH levels, and thereby promoting androgen secretion.

In summary, building upon the prior experience and advantages of the Yishen Tongluo Formula in treating male infertility, this study employed network toxicology to validate the toxic effects of cadmium chloride on male infertility, along with its primary mechanisms and action pathways, by identifying common targets between cadmium chloride and male infertility. The analysis yielded one common target for the Yishen Tongluo Formula in repairing cadmium-induced male infertility, twelve cadmium chloride-induced male infertility targets were identified, from which three key targets were selected: CFTR, SLC26A3, and SLC12A1. Through GO enrichment and KEGG pathway analysis, it was found that cadmium chloride primarily exerts functional effects via transmembrane transport, apical plasma membrane, and ion transmembrane transport activities, mainly impacting male reproductive function through the pancreatic secretion pathway. Supplementary indicators including GSH, TOS, seminiferous tubule diameter, Johnsen score, and immunohistochemical results of apoptosis and NF−κB pathway further validated the mechanism by which the Yishen Tongluo Formula restores cadmium−induced reproductive injury. The formula increased testicular coefficient in mice, reduced DFI, enhanced sperm concentration and total sperm motility, elevated SOD activity in testicular tissue, decreased MDA and nitric oxide (NO) levels, and elevate serum levels of luteinizing hormone (LH), follicle-stimulating hormone (FSH), and testosterone (T). This action regulates the endocrine system, mitigates oxidative stress damage to the testes, and facilitates the restoration of reproductive capacity impaired by cadmium toxicity.

Nevertheless, this study has several limitations. First, the protective effect of Yishen Tongluo Formula may also relate to anti-inflammatory and anti-apoptotic pathways, which needs further verification. Second, this study was performed only in mice, and clinical translation requires more evidence. In addition, we cannot rule out other signaling pathways involved in the therapeutic process (17, 18).

## 5 Summary and outlook

This study clarified that cadmium causes testicular toxicity and reproductive dysfunction by inducing oxidative stress, endocrine disorders and spermatogenic cell ultrastructural damage, while Yishen Tongluo Formula targets TP53 to reverse these injuries (14).Meanwhile, Yishen Tongluo Formula targets TP53 to resist oxidative stress, repair DNA damage and regulate endocrine function, thereby reversing cadmium-induced male reproductive injury. This leads to damage from oxidative stress, hormonal imbalances, and compromised DNA integrity, thereby posing a severe threat to male reproductive health. Network pharmacology predictions ind SLC12A1, and other key targets to disrupt pancreatic secretion pathways, thereby causing oxidative stress, hormonal dysregulation, and DNA integrity damage. This severely compromises male reproductive health. Network pharmacology predicted that the Yishen Tongluo Formula primarily repairs cadmium chloride-induced male infertility via the TP53 target; whereas conventional and modern medical treatments show clear limitations in addressing its long−term cumulative toxicity and multi−target damage. The Yishen Tongluo Formula stands as a practical exemplar of traditional Chinese medicine’s theory that ‘the kidneys govern reproduction’. Through its multi-component synergistic actions—such as antioxidant effects, hormone regulation, and DNA repair—it demonstrates a unique integrated therapeutic advantage encompassing detoxification, repair, and regulation. Nevertheless, systematic investigation remains necessary to elucidate the precise molecular mechanisms (including epigenetic regulatory networks and key signaling pathway interactions) and long-term clinical efficacy of this formula in addressing cadmium-induced male infertility. Future research should involve more extensive studies in cellular and animal models, examining the expression of relevant protein molecules within antioxidant signaling pathways to advance treatments for sperm DNA damage in male infertility.

## Data Availability

The original contributions presented in the study are included in the article/supplementary material. Further inquiries can be directed to the corresponding author/s.
